# Coding-complete genome sequence of a divergent member of the genus *Gordisvirus* detected in *Sabethes* (*Peytonulus*) *undosus* Coquillet mosquitoes (Diptera: Culicidae) from Brazil

**DOI:** 10.1128/mra.01275-24

**Published:** 2025-04-28

**Authors:** Daniel Dias, Bruna Nascimento, Ana Cruz, Sandro Silva, Lúcia Reis, Fábio Silva, Lucas Silva, Sâmia Silva, Durval Vieira, Roberto Brandão, José Junior, Alessandra Santos, Hanna Reis, Joaquim Neto

**Affiliations:** 1 Graduate Program in Parasitary Biology in the Amazon Region, Center of Biological and Health Sciences, State University of Pará, Belém, Brazil; 2 Department of Arbovirology and Hemorrhagic Fevers, Evandro Chagas Institute, Ministry of Health, Ananindeua, Brazil; Katholieke Universiteit Leuven, Leuven, Belgium

**Keywords:** *Gordisvirus*, metagenomics, mosquitoes

## Abstract

We report the complete genome sequence of a divergent member of the genus *Gordisvirus* (family *Xinmoviridae*, order *Mononegavirales*), obtained through metagenomic sequencing of *Sabethes* (*Peytonulus*) *undosus* Coquillett mosquitoes in the Brazilian Amazon. Phylogenetic analyses confirmed its classification. The genome comprises 12,150 nucleotides and encodes five open-reading frames.

## ANNOUNCEMENT

*Xinmoviridae* is a family of negative-sense RNA viruses with genomes of 9 to 14 kilobases that typically infect arthropods (1). Virome studies in mosquitoes have expanded the knowledge about Xinmovirids, leading to the identification of several novel viruses (2, 3). Here, we report the coding-complete genome sequence of a divergent member of the genus *Gordisvirus*, recovered through metagenomic sequencing of a pool of ten female *Sabethes* (*Peytonulus*) *undosus* Coquillet collected in 2022 in Belém, Pará, Brazilian Amazon.

The pool containing whole insect bodies was homogenized in 500 µL of Dulbecco’s Phosphate Buffered Saline (Gibco, Massachusetts, USA), with 2% penicillin and streptomycin (Sigma-Aldrich, Massachusetts, USA; Cat.No. P4333), 1% Amphotericin B (Sigma-Aldrich; Cat.No. A2942), 5% fetal bovine serum (LGC Biotecnologia, São Paulo, Brasil; Cat.No.10-bio500), and a 3 mm tungsten bead (Qiagen, Hilden, Germany) using TissueLyser II (Qiagen) at 25 Hz for 1 min. Viral RNA was extracted using the QIAamp Viral RNA kit (Qiagen). cDNA synthesis was performed with the SuperScript (Agilent Technologies, California, USA) and NEBNext (New England Biolabs, USA) kits. Libraries were prepared with the SureSelectQXT kit (Agilent) and sequenced by the paired-end method on the NextSeq 550 platform (Illumina, California, USA) using the NextSeq 500/550 High Output kit v.2.5 (300 cycles).

Quality control of reads was performed using Fastp v.0.23.4 (4). *De novo* assembly was conducted using MEGAHIT v.1.2.9 (5). Contigs were compared to the RefSeq viral database (https://ftp.ncbi.nlm.nih.gov/refseq/release/viral/) using DIAMOND v.2.1.10 (6) and inspected with MEGAN v.25.10 (7). Open reading frames (ORFs) were predicted using NCBI ORFfinder (https://www.ncbi.nlm.nih.gov/orffinder/) and annotations were manually performed by comparison with other *Xinmoviridae* using BLASTx (https://blast.ncbi.nlm.nih.gov/Blast.cgi). Putative transmembrane domains were identified using TMHMM (http://www.cbs.dtu.dk/services/TMHMM/). The RNA-dependent RNA polymerase sequence was compared with other *Xinmoviridae* available at NCBI using ClustalW v.2.1 (8). Phylogeny was inferred by Maximum Likelihood with 1000 bootstrap replicates using IQ-TREE v2.3.6 (9), and topologies were visualized with FigTree v.1.4.4 (10). All tools were run with default parameters.

**Fig 1:**
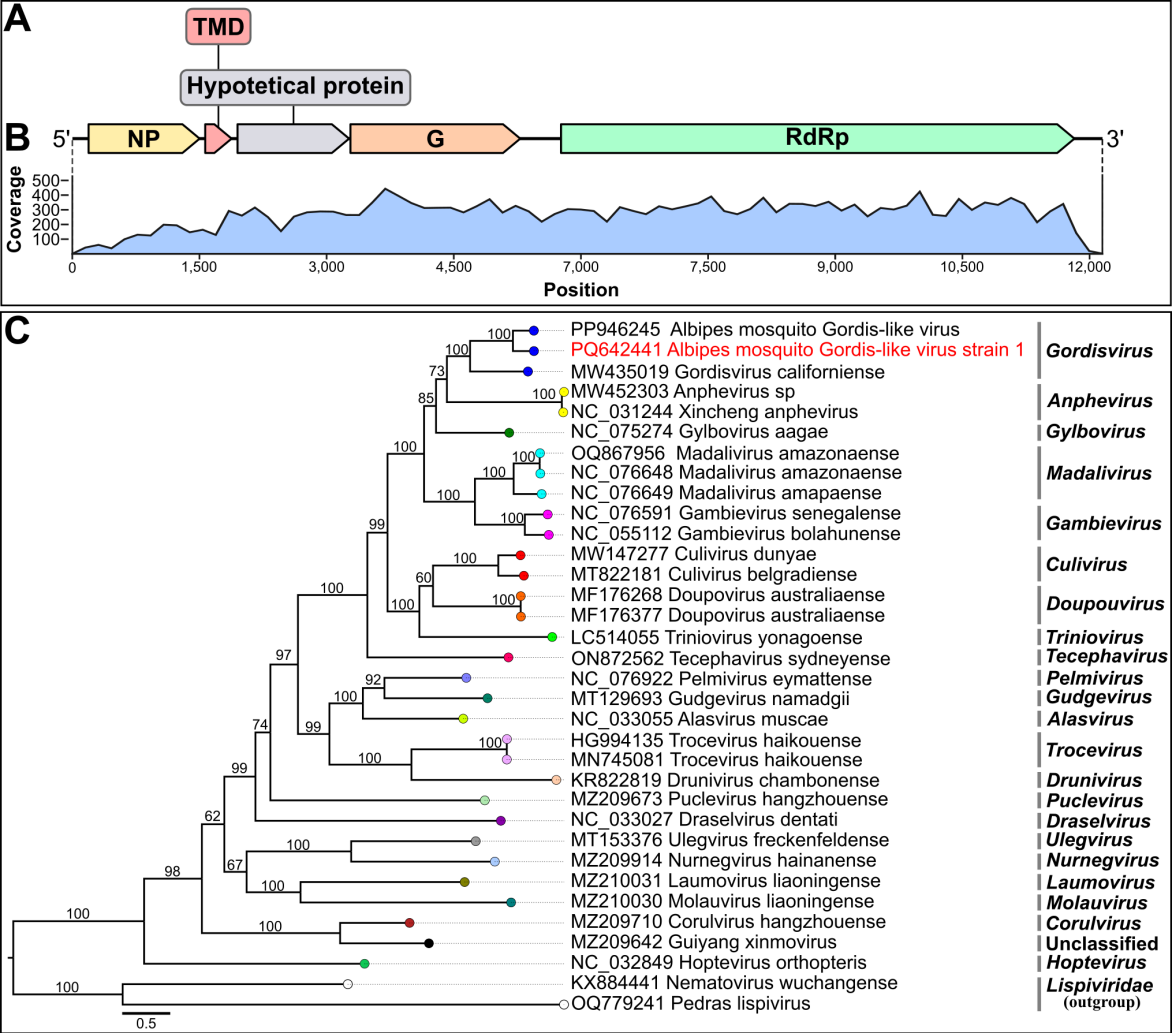
(**A**) Genome organization of Albipes mosquito Gordis-like virus strain 1. Predicted open reading frames are indicated by arrows. (**B**) Genome coverage is represented by the blue graph. The untranslated terminal regions (UTRs) were not determined due to low read coverage in these regions. (**C**) Phylogenetic relationship between the Albipes mosquito Gordis-like virus strain 1 RdRp sequence (highlighted in red) and sequences of viruses from the *Xinmoviridae* family available in the NCBI database. The phylogenetic tree was inferred using the maximum likelihood method with the Q.pfam +F + R6 substitution model for amino acids and statistical support of bootstrap with 1000 replicates. The tree was rooted using the midpoint method implemented in FigTree v.1.4.4. The scale bar indicates amino acid substitutions per site. Bootstrap support values are shown at each node, and GenBank accession numbers are listed to the left of the virus names.

The sequencing yielded 15,694,292 reads of which 14,536,750 remained after quality control. The recovered contig was 12,150 bp long, with 24,834 mapped reads, GC content of 42.52%, and an average coverage of 272×. This contig contained five ORFs organized similarly to those observed in the *Mononegavirales*, in the following 5′−3′ terminal gene order: nucleoprotein (NP; 1,311 nt), small transmembrane protein (STM; 312 nt), hypothetical protein (1,317 nt), glycoprotein (G; 2,004 nt), and RNA-dependent RNA polymerase (RdRp; 6,057 nt) (Fig. 1A). The UTR (5′−3′) region was not determined due to low coverage (Fig. 1B). The RdRp sequence was closely related to the Albipes mosquito Gordis-like virus (GenBank accession XGU11772), sharing 72.83% (amino acids) and 65.89% (nucleotides) identity. Phylogenetic analysis showed that the obtained sequence clustered with other members of the *Gordisvirus* genus, with 100% bootstrap support (Fig. 1C). According to ICTV, an amino acid identity of <66% in the RdRp region is a criterion for species demarcation within the *Gordisvirus* genus (1). Therefore, we propose that the recovered genome could be classified as a divergent member of the genus *Gordisvirus,* tentatively named “Mosquito Albipes Gordis-like strain 1”.

## Data Availability

The Albipes mosquito Gordis-like virus strain 1 genome sequence has been deposited in GenBank under accession number PQ642441. The raw data have been deposited in the NCBI Sequence Read Archive database under accession number SRX26805044.
